# Artificial Intelligence-Derived Computed Tomography Phenotyping for Cardiovascular Risk Stratification in Transcatheter Mitral Valve Replacement

**DOI:** 10.3390/jcm15197400

**Published:** 2026-09-23

**Authors:** Liliane Zillner, Tillmann Kerbel, Paul Sautner, Mirjam G. Wild, Michaela M. Hell, Elmar W. Kuhn, Tanja K. Rudolph, Thomas Walther, Lenard Conradi, Andreas Zierer, Francesco Maisano, Marco Russo, Fabrizio Rosati, Andrea Colli, Miguel Piñón, David Reineke, Gaby Aphram, Christophe Dubois, Jörg Hausleiter, Ralph Stephan von Bardeleben, Iuliana Coti, Christian Loewe, Daniel Zimpfer, Georg Heinze, Martin Andreas

**Affiliations:** 1 Department of Cardiac and Thoracic Aortic Surgery, Medical University of Vienna, 1090 Vienna, Austria; 2 Medizinische Klinik I, LMU University Hospital, 81377 Munich, Germany; 3 Department of Cardiology, University Medical Center Mainz, 55131 Mainz, Germany; 4 Department of Cardiothoracic Surgery, Heart Center, University Hospital Cologne, 50937 Cologne, Germany; 5 Department of Cardiology, Heart and Diabetes Center North Rhine-Westphalia, Ruhr University Bochum, 44801 Bad Oeynhausen, Germany; 6 Department of Cardiac Surgery, University Hospital Frankfurt, 60590 Frankfurt, Germany; 7 Department of Cardiac Surgery, Heart Center, University Hospital Cologne, 50937 Cologne, Germany; 8 Department for Cardiac, Vascular and Thoracic Surgery, Kepler University Hospital Linz, 4020 Linz, Austria; 9 Valve Center, IRCCS Ospedale San Raffaele, University Vita-Salute San Raffaele, 20132 Milan, Italy; 10 Department of Cardiac Surgery and Heart Transplantation, Azienda Ospedaliera San Camillo Forlanini, 00152 Rome, Italy; 11 Division of Cardiac Surgery, Spedali Civili di Brescia, University of Brescia, 25121 Brescia, Italy; 12 Department of Surgical, Medical and Molecular Pathology and Critical Care Medicine, University of Pisa, 56126 Pisa, Italy; 13 Servicio Cirugía Cardíaca, Hospital Álvaro Cunqueiro, 36312 Vigo, Spain; 14 Department of Cardiac Surgery, Inselspital University Hospital Bern, 3010 Bern, Switzerland; 15 Department of Cardiovascular and Thoracic Surgery, Cliniques Universitaires Saint-Luc, 1200 Brussels, Belgium; 16 Department of Cardiovascular Medicine, University Hospital Leuven, 3000 Leuven, Belgium; 17 Department of Cardiovascular Sciences, KU Leuven, 3000 Leuven, Belgium; 18 German Centre for Cardiovascular Research, Munich Heart Alliance, 80336 Munich, Germany; 19 Division of Cardiovascular and Interventional Radiology, Department of Biomedical Imaging and Image-Guided Therapy, Medical University of Vienna, 1090 Vienna, Austria; 20 Institute of Clinical Biometrics, Center for Medical Data Science, Medical University of Vienna, 1090 Vienna, Austria; 21 Division of Cardiac Surgery, Department of Surgery, Medical University Graz, 8010 Graz, Austria

**Keywords:** transcatheter mitral valve replacement, artificial intelligence, computed tomography, segmentation, phenotyping, body-composition, Tendyne^TM^ valve

## Abstract

**Background:** Despite high procedural success of transcatheter mitral valve replacement (TMVR), mortality remains substantial, and conventional risk scores inadequately reflect the specific risk profile of this high-risk population. Artificial intelligence (AI)-derived analysis may enable TMVR-specific risk assessment using routinely acquired computed tomography (CT). **Methods:** In a retrospective European multicenter registry, AI-based segmentation of harmonized real-world pre-procedural cardiac CT with variable thoracic and upper-abdominal coverage was applied in patients undergoing TMVR with the Tendyne^TM^ system (134 patients; 346 CT-derived variables retained for high-dimensional screening). One-year cardiovascular mortality was assessed using Cox proportional hazards models with Benjamini–Hochberg false discovery rate correction. STS- and EuroSCORE II-adjusted analyses and a targeted coronary-ventricular coupling analysis were performed. **Results:** In the STS-adjusted Cox analysis, nine AI-derived CT parameters met the 10% FDR discovery threshold for cardiovascular mortality, spanning skeletal (n = 3), muscular (n = 1), and cardiopulmonary phenotypes (n = 5), whereas three parameters met this threshold in CT-only analyses. Coronary–ventricular coupling, defined as coronary artery volume relative to left ventricular end-diastolic volume, was significantly associated with lower 1-year cardiovascular mortality in the CT-only analysis (HR per IQR 0.47, 95% CI 0.22–1.00; *p* = 0.049) and after EuroSCORE II adjustment (HR 0.47, 95% CI 0.22–0.99; *p* = 0.046), while the STS-adjusted estimate remained directionally consistent but did not reach statistical significance (HR 0.49, 95% CI 0.23–1.05; *p* = 0.067). **Conclusions:** AI-based CT phenotyping identifies imaging-derived markers associated with cardiovascular mortality beyond information captured by conventional surgical risk scores in TMVR patients. Coronary-ventricular coupling emerges as a promising automated CT-derived imaging marker with a biologically plausible pathophysiological basis, linking coronary arterial volume to ventricular size in mitral valve disease.

## 1. Introduction

Transcatheter mitral valve replacement (TMVR) is an evolving treatment option for patients with severe symptomatic mitral regurgitation who are ineligible for surgical mitral valve replacement or transcatheter edge-to-edge repair. In this context, the Sapien M3 valve (Edwards, CE 2025; ~100 implants) and the transapical Tendyne system (Abbott, CE 2020; FDA 2025; ~2000 implants) have shown promising results [1,2,3]. While transseptal TMVR is expected to become the predominant approach, as suggested by improved outcomes in valve-in-valve and valve-in-ring procedures, transapical TMVR will remain essential in selected patients due to specific anatomical constraints, enhanced procedural control, and apical anchoring in severe mitral annular calcification [4,5]. Given the limited TMVR evidence base, insights from transapical TMVR may also improve future transseptal strategies.

Despite high procedural success rates after Tendyne TMVR (97%), all-cause (39%) and cardiovascular mortality (34%) at two years remain substantial [6]. This highlights the need for improved risk prediction beyond conventional scores such as the Society of Thoracic Surgeons (STS) score and EuroSCORE II, which were not designed for TMVR and show limited discriminatory performance in this population.

Several studies have emphasized the prognostic relevance of anatomical risk markers. Kerbel et al. identified thin apical myocardium, and Zillner et al. small left-ventricular end-diastolic diameter index (LVEDDi), as predictors of periprocedural mortality [7,8]. Furthermore, a large annulus might predict cardiac death at one year [9,10]. Currently, as TMVR development shifts towards transseptal devices, CT-based anatomy may be especially relevant for predicting outcomes beyond 30-days, as early mortality has largely been driven by transapical access-related bleeding. Pre-procedural CT may capture quantitative structural information beyond the selected clinical and echocardiographic variables incorporated in conventional surgical risk scores.

Recent advances in artificial intelligence (AI) and CT imaging have enabled automated screening and sizing, streamlining TMVR planning [11]. Beyond cardiac assessment, AI-based CT analysis allows objective quantification of body composition and extracardiac features, including adipose tissue, vascular, and skeletal parameters; however, such comprehensive CT-based phenotyping has not yet been applied with regard to the highly selected TMVR population. Unlike conventional surgical scores or isolated anatomical measurements, comprehensive automated CT phenotyping can simultaneously quantify multiple cardiac and extracardiac domains from imaging studies already acquired for procedural planning, providing an objective framework for identifying risk signals not represented by a single clinical or anatomical variable.

Frailty is an independent predictor of adverse outcomes after transcatheter valve interventions, yet current surgical risk models do not formally incorporate multidimensional frailty [12,13,14,15]. While clinical frailty assessments are often subjective and inconsistently applied, CT-derived body composition metrics offer objective and reproducible surrogates of physiological reserve that can be automatically extracted from routine TMVR planning CTs. Accordingly, this study evaluates whether AI-derived CT variables provide incremental prognostic value beyond established clinical risk scores in this evolving TMVR patient cohort with limited available data.

## 2. Materials and Methods

### 2.1. Ethical Statement

The TENDER registry was approved by the ethics committees of all participating centers, including the Ethics Committee of the Medical University of Vienna (approval No. 1434/2022). Data were anonymized, and all patients provided written informed consent for data use.

### 2.2. Design and Participants

This retrospective observational study included patients from 16 European centers who underwent TMVR with the Tendyne^TM^ system and were enrolled in the TENDER registry. The TENDER registry is an investigator-initiated observational registry registered on ClinicalTrials.gov (NCT04898335). All 134 patients underwent contrast-enhanced cardiac ECG-gated 4D-CT as part of pre-procedural assessment. Imaging protocols varied according to local clinical practice. Scans were acquired for clinical planning and typically covered the heart and thorax, reflecting routine real-world imaging rather than dedicated whole-body CT (Figure 1). The multiphase acquisitions additionally enabled volumetric characterization of cardiac structures across the cardiac cycle. Clinical data, including baseline characteristics, comorbidities, and STS-PROM, were obtained from planning documentation and procedural reports. All AI-based image analyses were performed retrospectively after the TMVR procedures; the derived CT measurements and study analyses were not available to treating operators and therefore did not influence patient selection, procedural planning, or procedural conduct.

AI-based CT analysis quantified body composition parameters (bone, skeletal muscle, intramuscular, subcutaneous, visceral, pericardial, and epicardial adipose tissue), as well as organ dimensions, aortic calcification, and vascular measurements. Segmentation was performed using the Body and Organ Analysis (BOA) software v1.0.2 (University Medicine Essen, Essen, Germany; https://github.com/UMEssen/Body-and-Organ-Analysis, accessed on 15 September 2026) [16] and Comp2Comp (Stanford Machine Intelligence in Medical Imaging [MIMI] Lab, Stanford, CA, USA; https://github.com/StanfordMIMI/Comp2Comp, accessed on 15 September 2026) [17]. No new AI model was trained in this study. Instead, the previously developed automated AI segmentation models BOA and Comp2Comp were applied retrospectively to the harmonized pre-procedural CT scans to generate quantitative anatomical phenotypes, which were subsequently evaluated using the statistical analyses described below.

The largest suitable three-dimensional scan was selected for body and organ analysis using measured z-axis coverage, while complete cardiac acquisitions were retained for phase-resolved heart segmentation. All selected volumes were harmonized with a 1 mm full-width-at-half-maximum low-pass filter and resampled to a 1 mm isotropic grid. End-diastole and end-systole were defined from the measured maximum and minimum segmented LV cavity volumes. Detailed imaging selection, harmonization and quality control are provided in Supplementary Methods S2. This harmonization step was applied uniformly across centers to reduce technical variability related to scanner- and reconstruction-specific differences and to support analysis of heterogeneous real-world multicenter CT data.

### 2.3. Study Endpoints and Statistical Analysis

One-year cardiovascular mortality was the primary endpoint. Cardiovascular mortality was classified according to Mitral Valve Academic Research Consortium (MVARC) endpoint definitions, as detailed in Supplementary Methods S2 [18].

The high-dimensional discovery analysis was restricted to automated CT variables. Features were required to have ≥75% completeness; right-skewed measures were log/log1p transformed, and deterministic outcome-blind Spearman pruning at |ρ| ≥ 0.95 reduced redundancy, leaving 346 features. Cox models were fitted as CT-only, CT + STS and CT + EuroSCORE II models, with HRs scaled per IQR. BH-FDR correction was applied separately within each endpoint and model family; q < 0.10 was considered statistically significant in the exploratory discovery analysis. Proportional-hazards assumptions were assessed using Schoenfeld residuals.

Thus, AI was used to convert the CT images into quantitative phenotypes, whereas feature eligibility was determined outcome-blind according to data availability and redundancy criteria before each retained variable was tested for association with mortality.

In an additional post hoc sensitivity analysis, baseline MR grade was evaluated as an ordinal variable. Spearman correlations quantified associations between baseline MR grade and the nine CT features identified in the STS-adjusted cardiovascular-mortality discovery analysis. Each of these nine features was then re-evaluated in a Cox model adjusted for both STS-PROM and baseline MR grade; Benjamini–Hochberg correction was applied across this targeted nine-feature sensitivity set.

A separate targeted analysis evaluated coronary-ventricular coupling. The primary coupling measure was defined as coronary artery volume (CAV) divided by left ventricular end-diastolic volume (LV-EDV), corresponding to the maximal segmented LV cavity volume. LV-EDV was selected as the primary denominator because it reflects maximal ventricular chamber size and is pathophysiologically relevant to the chronic volume loading and ventricular dilatation characteristic of mitral regurgitation. This ratio combined two biologically related CT-derived measures already available from the automated phenotyping pipeline. Alternative normalization using LV end-systolic and myocardial volumes was assessed in sensitivity analyses. CAV and the individual cardiac-volume components were additionally examined to contextualize the coupling signal. Cox models were fitted as CT-only, STS-adjusted, and EuroSCORE II-adjusted models, with HRs expressed per IQR. For the primary CAV/LV-EDV association, nominal *p*-values are reported directly. BH-FDR-adjusted q-values across the broader targeted coronary/coupling family were retained as a supportive multiplicity analysis. Discrimination was assessed using C-indices with bootstrap-based internal validation and paired bootstrap comparisons; spline models assessed nonlinearity.

For complementary outcome-independent domain analysis, the 346 CT features retained after preprocessing were summarized by principal component analysis (PCA) and evaluated using the same Cox framework. Elastic-net Cox regression with bootstrap validation assessed whether a stable multidomain CT score could be derived. Full PCA, calibration, competing-risk, validation, and sensitivity procedures are reported in Supplementary Methods S2.

During the preparation of this work, the authors used AI for English-language editing and assistance with the graphical abstract. The authors subsequently reviewed and edited all content and take full responsibility for the final article and conclusions.

## 3. Results

### 3.1. Baseline Parameters

Baseline characteristics are summarized in Table 1 and reflect a typical TMVR population characterized by advanced age, frailty, and severe symptomatic mitral regurgitation. Patients were elderly (median age 77 years) and at high procedural risk, with a median STS score of 6.0% (EuroSCORE II of 6.3%). The majority presented with advanced heart failure symptoms (NYHA III–IV: 85.9%) and severe MR (grade ≥ 3 in 95.5%), with comparable proportions of primary and secondary MR. The high burden of comorbidities, impaired renal function, and reduced functional reserve underscores the relevance of CT-based phenotyping, including cardiopulmonary and musculoskeletal parameters, for refined risk stratification in this vulnerable TMVR cohort.

### 3.2. Causes of Death

Cardiovascular mortality within one year was observed in 21 of 105 patients with evaluable cardiovascular-mortality status (20.0%). Within 30 days, 9 of 125 patients with evaluable all-cause mortality status died (7.2%), including 7 cardiovascular deaths among 114 patients with evaluable cardiovascular-mortality status (6.1%; 2 with LVOT obstruction, 1 ventricular rupture with tamponade, 1 cardiogenic shock, and 3 other cardiovascular causes, 1 associated with sepsis) and 2 non-cardiovascular deaths due to sepsis. At one year, a total of 34 all-cause deaths were recorded among 114 patients with evaluable all-cause mortality status (29.8%), including 14 cardiovascular deaths occurring beyond 30 days (4 due to heart failure, 2 stroke, 6 other cardiovascular causes, 1 thromboembolic intestinal ischemia, and 1 ventricular tachycardia) and 11 non-cardiovascular deaths beyond 30 days, mainly due to sepsis, multi-organ failure, or neurological causes.

### 3.3. AI-Derived CT Markers for 1-Year Cardiovascular Mortality

From an initial set of 5729 numeric CT-derived variables, 346 features were retained after preprocessing, completeness filtering, and redundancy reduction (Supplementary Methods S2, Figure S1). In the STS-adjusted Cox analysis, nine AI-derived CT parameters met the 10% FDR discovery threshold for 1-year cardiovascular mortality. These comprised skeletal features (total left ninth-rib volume, left tenth-rib volume, and right fourth-rib minimum attenuation), cardiopulmonary features (lung/tracheobronchial attenuation heterogeneity, left and right upper-lobe volumes, lung-vessel volume, and pleural-effusion volume), and one muscular feature. The strongest association was observed for total left ninth-rib volume (HR per IQR 5.56, 95% CI 2.06–14.99; q = 0.095). In the CT-only analysis, three features remained below the 10% FDR threshold: lung/tracheobronchial attenuation heterogeneity, total left ninth-rib volume, and left upper-lobe volume. No CT feature met the 10% FDR threshold for 1-year all-cause mortality or in the EuroSCORE II-adjusted high-dimensional analyses (Figure 2; Table 2, Supplementary Results S1, Table S1).

In the post hoc MR-severity sensitivity analysis, baseline MR grade itself was associated with both 1-year all-cause mortality (HR per one-grade increase 5.56, 95% CI 1.71–18.07; *p* = 0.004) and cardiovascular mortality (HR 9.64, 95% CI 1.31–70.71; *p* = 0.026). These associations remained significant after adjustment for STS-PROM (all-cause mortality: HR 5.15, 95% CI 1.58–16.81; *p* = 0.007; cardiovascular mortality: HR 10.12, 95% CI 1.36–75.13; *p* = 0.024). Correlations between baseline MR grade and the nine STS-adjusted CT discovery features were weak to moderate (absolute Spearman rho 0.17–0.35). After additional adjustment for both STS-PROM and baseline MR grade, all nine features remained below q < 0.10 within this targeted sensitivity set. The strongest retained associations were observed for total left ninth-rib volume (HR per IQR 6.07, 95% CI 1.80–20.43; q = 0.032) and total left tenth-rib volume (HR 3.76, 95% CI 1.43–9.85; q = 0.032). Detailed correlations and MR-adjusted Cox results are provided in Supplementary Results S1, Table S5.

### 3.4. Coronary–Ventricular Coupling: Comparison with Clinical Scores

Coronary-ventricular coupling was evaluated separately using the primary definition of CAV/LV-EDV. Coronary–ventricular coupling was evaluated separately using the primary definition of CAV/LV-EDV. For 1-year cardiovascular mortality, higher coupling was significantly associated with lower mortality in the CT-only model (HR per IQR 0.47, 95% CI 0.22–1.00; *p* = 0.049; n = 86, 17 events) and after adjustment for EuroSCORE II (HR 0.47, 95% CI 0.22–0.99; *p* = 0.046; n = 83, 17 events). The STS-adjusted estimate remained directionally consistent but did not reach statistical significance (HR 0.49, 95% CI 0.23–1.05; *p* = 0.067; n = 79, 15 events) (Table 3).

Coupling alone showed numerically higher discrimination than STS (C-index 0.647 vs. 0.619) and EuroSCORE II (0.647 vs. 0.635), although paired bootstrap comparisons did not demonstrate significant differences. Addition of coupling numerically increased discrimination from 0.619 to 0.635 for STS and from 0.635 to 0.658 for EuroSCORE II. Bootstrap internal validation yielded an optimism-corrected C-index of 0.620 for coupling alone; the corresponding corrected C-indices were 0.586 for STS and 0.620 for EuroSCORE II.

CAV alone showed a concordant inverse association, and alternative normalization of CAV to LV end-systolic or myocardial end-diastolic/end-systolic volumes yielded directionally consistent estimates (Supplementary Results S1, Tables S4 and S5). No corresponding association was observed for 1-year all-cause mortality. Natural-spline analyses showed no evidence that the relationship between CAV/LV-EDV and cardiovascular mortality deviated from linearity.

### 3.5. Outcome-Independent Domain Phenotypes and Multidomain Modelling

In the complementary outcome-independent PCA, vascular and multiorgan phenotypes were associated with 1-year cardiovascular mortality in CT-only models (Vascular PC3: HR per SD 1.77, 95% CI 1.18–2.66; q = 0.089; Other Organs PC2: HR 0.70, 95% CI 0.54–0.91; q = 0.089), but neither remained below the 10% FDR threshold after adjustment for STS or EuroSCORE II. Penalized multidomain modelling did not yield a stable CT-based prediction score. Detailed PCA and bootstrap-validation results are provided in Supplementary Results S1, Table S6.

## 4. Discussion

### 4.1. AI-Derived Risk Phenotyping in TMVR with Harmonized CT

A key finding of the present study is that AI-derived CT phenotyping, both with and without STS adjustment, identified imaging features associated with cardiovascular mortality within 1 year, whereas no CT-derived feature met the 10% FDR discovery threshold for all-cause mortality (Figure 3). This endpoint-specific distinction is noteworthy, as the identified CT phenotypes included predominantly structural characteristics spanning skeletal, muscular, and cardiopulmonary domains and might therefore intuitively be expected to reflect broader vulnerability rather than predominantly cardiovascular death. However, cardiovascular deaths in our cohort comprised heart failure progression, thromboembolic events, malignant arrhythmias, stroke, and mechanical complications, pathophysiological processes that may plausibly be reflected by CT-derived phenotyping, whereas infection- or sepsis-related deaths were less likely to be captured by pre-operative structural imaging. Although several of the identified features may overlap conceptually with imaging correlates of frailty or systemic vulnerability, frailty itself was not directly assessed in this study [19,20]. In contrast to these extracardiac structural phenotypes, coronary-ventricular coupling represents a fully automated cardiac imaging marker integrating coronary arterial volume with left ventricular size. Higher coupling was associated with lower 1-year cardiovascular mortality in the CT-only and EuroSCORE II-adjusted analyses, with a directionally consistent estimate after STS adjustment. Collectively, these findings support a predominantly cardiovascular rather than all-cause signal of automated CT phenotyping in patients with advanced mitral regurgitation undergoing transapical TMVR. The multicenter harmonization workflow is particularly relevant in this setting because TMVR cohorts are necessarily assembled across centers with heterogeneous scanners and reconstruction protocols; standardized filtering and isotropic resampling were used to reduce this technical heterogeneity before automated phenotyping.

The additional MR-severity sensitivity analysis suggests that the identified CT phenotypes are not simply surrogates of baseline valvular severity. Their correlations with MR grade were weak to moderate, and the nine STS-adjusted discovery features retained associations with cardiovascular mortality after further adjustment for MR grade within the targeted sensitivity analysis. Because this analysis was post hoc and it involved limited event numbers and few patients with lower MR grades, it should be interpreted as supportive rather than confirmatory and does not establish MR-specific mechanisms.

### 4.2. Skeletal and Muscular Domain

Skeletal attenuation and volumetric markers among the CT-derived features associated with cardiovascular mortality included left ninth- and tenth-rib volume and minimum attenuation of the right fourth rib. These findings suggest these CT-derived measures captured morphological skeletal indicators potentially related to cardiovascular vulnerability not reflected in conventional risk scores. In accordance with our findings, thoracic bone attenuation on routine CT has previously been linked to bone mineral density and skeletal health [21]. In our cohort, lower minimum rib attenuation was associated with higher cardiovascular mortality risk, which may reflect lower bone mineralization, although bone mineral density was not directly measured [22]. In contrast, higher rib volumes, including left ninth-rib volume as the strongest association across the analyzed domains, may reflect age-related skeletal remodeling and degenerative bone enlargement, as previously shown in elderly patients [23]. Within the muscular domain, higher intramuscular adipose tissue (IMAT) was associated with cardiovascular mortality. Increased muscular fat infiltration, or myosteatosis, is an established imaging marker of impaired muscle quality and has been associated with frailty and adverse outcomes in older populations [24]. Thus, in our high-risk elderly TMVR population, lower skeletal attenuation, larger bone volume, and increased IMAT may reflect different aspects of musculoskeletal remodeling associated with cardiovascular vulnerability, although the underlying biological mechanisms remain uncertain.

### 4.3. Cardiopulmonary Domain

Within the cardiopulmonary domain, larger lung volume (left and right upper-lobe volume), lung-vessel volume, pleural-effusion volume, and lung/tracheobronchial attenuation heterogeneity were associated with cardiovascular mortality. Increased upper-lobe volumes may partly reflect underlying pulmonary hyperinflation, as seen in emphysema, where increasing disease severity is accompanied by substantially increased regional lung air volumes [25]. Furthermore, lung/tracheobronchial attenuation heterogeneity may reflect structural pulmonary remodeling associated with underlying lung disease [26]. Increased lung-vessel volume may additionally reflect alterations in pulmonary vascular and hemodynamic burden, as quantitative CT-derived pulmonary vessel volumes have previously been associated with pulmonary hypertension phenotypes [27]. Finally, pleural-effusion volume may represent an imaging correlate of congestion in patients with advanced cardiac disease. Thus, these pulmonary and thoracic CT phenotypes may capture different aspects of cardiopulmonary vulnerability and congestion in this high-risk TMVR population.

### 4.4. Coronary–Ventricular Coupling as New Automated Predictor

The coronary-ventricular coupling index, calculated as coronary artery volume (CAV) over LV end-diastolic volume, may primarily refine the prognostic value of CAV by accounting for the size of the ventricle it supplies. In this sense, a lower index may suggest that coronary vascular capacity is insufficient relative to LV enlargement and remodeling. This interpretation is conceptually related to previous CCTA-based studies that linked a lower CAV-to-LV-myocardial-mass index to long-term MACE in a cohort with suspected coronary artery disease [28]. Furthermore, the CAV-to-LV-mass coupling index was previously reported to predict adverse outcomes in TAVR populations. In aortic stenosis, pressure overload induces myocardial hypertrophy, and a reduced coronary artery volume relative to LV mass may indicate insufficient coronary supply for the hypertrophied myocardium [29]. However, in advanced MR, the ventricle tends to dilate and increase in size. Accordingly, our index relates coronary artery volume to LV enlargement rather than to myocardial mass, although alternative normalization to myocardial end-diastolic and end-systolic volumes showed consistent associations in sensitivity analyses. In the primary CAV/LV-EDV analysis, the inverse association was statistically significant in the CT-only model (*p* = 0.049) and after EuroSCORE II adjustment (*p* = 0.046), whereas the STS-adjusted estimate remained directionally consistent but did not reach statistical significance (*p* = 0.067). Importantly, particularly in elderly patients, ventricular dilation may coexist with increased myocardial wall thickness due to age-related remodeling. Thus, coronary-ventricular coupling represents a promising candidate for integration into a future automated CT-based risk score in TMVR.

### 4.5. Limitations

Given the limited statistical power and low event rates, these findings should be considered hypothesis-generating and warrant further prospective evaluation. In addition, this study did not include a separate comparator population with another valvular disease; therefore, disease specificity of the identified extracardiac phenotypes cannot be established from the present analysis. Evaluation in independent TMVR cohorts and in other valvular populations, including tricuspid valve disease, will be important to assess generalizability, disease specificity, and the potential role of these CT phenotypes in future automated risk models.

## 5. Conclusions

In this multicenter TMVR study, AI-derived CT phenotyping identified markers across the skeletal, cardiopulmonary, and muscular domains associated with cardiovascular mortality. Importantly, these signals emerged from routinely acquired real-world cardiac CT with variable extracardiac coverage, highlighting the potential to extract clinically relevant risk information from existing imaging without additional radiation exposure, while multiphase cardiac CT enables precise volumetric characterization of cardiac structures across the cardiac cycle. Coronary–ventricular coupling emerged as a promising automated cardiac imaging marker, with statistically significant associations in the CT-only and EuroSCORE II-adjusted analyses, a directionally consistent STS-adjusted estimate, and consistent findings across alternative normalization strategies. This frontier analysis supports the development of an automated CT-based risk score as larger cohorts become available.

## Figures and Tables

**Figure 1:**
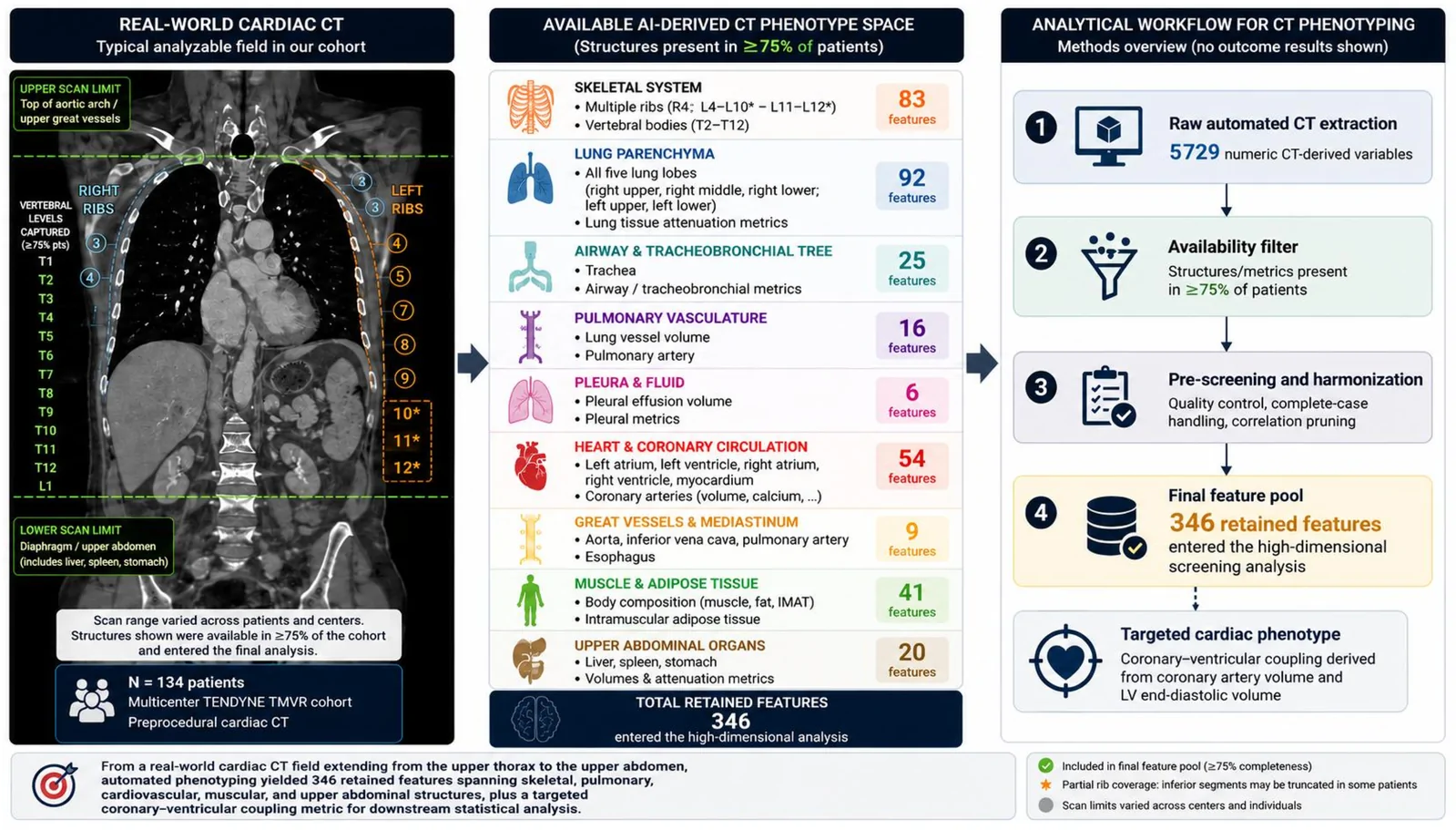
AI-derived CT phenotyping from routine, real-world cardiac CT for cardiovascular mortality in TMVR. * Partial rib coverage: inferior segments may be truncated in some patients.

**Figure 2:**
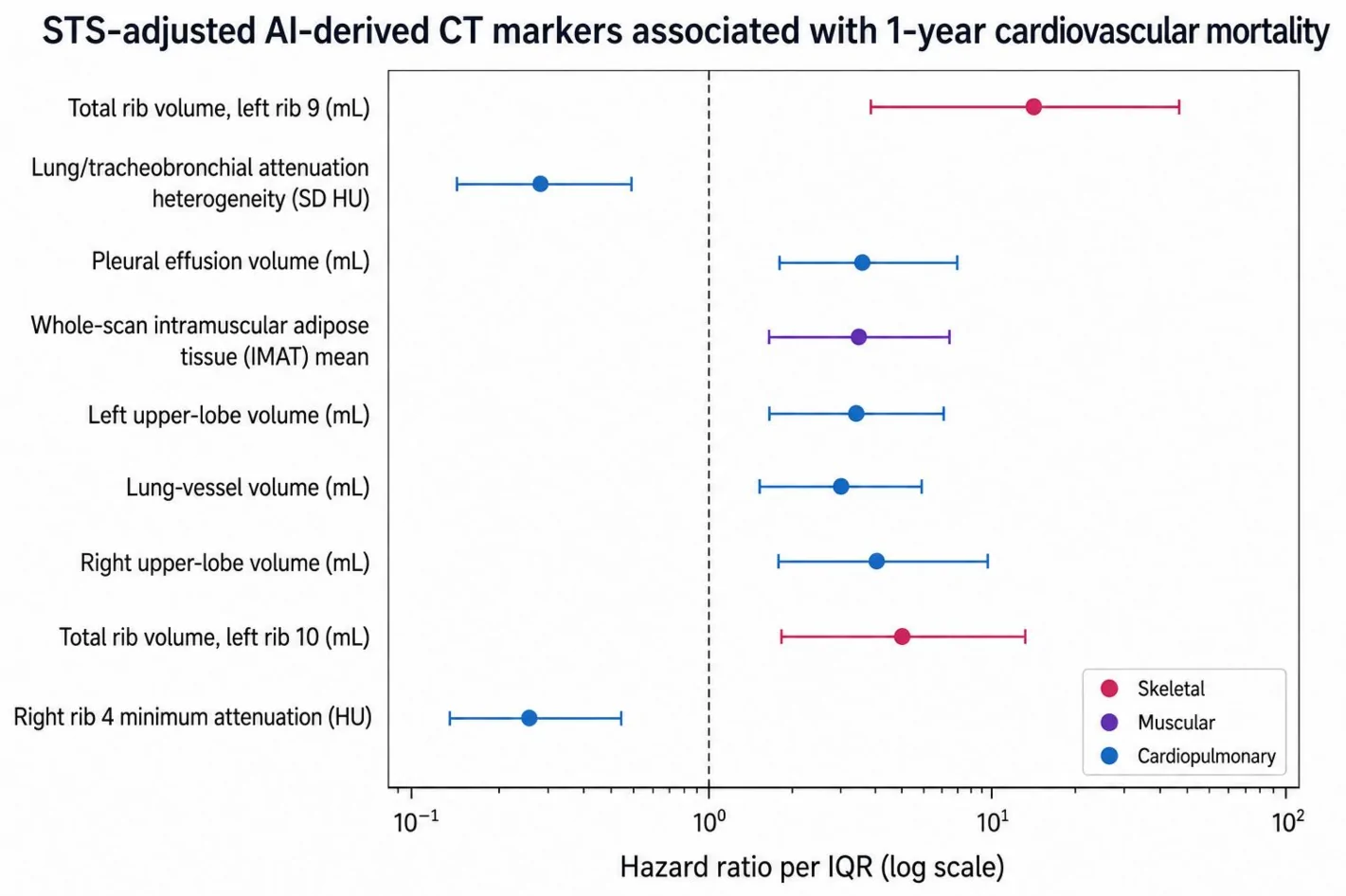
AI-derived CT Markers for Cardiovascular Mortality in TMVR.

**Figure 3:**
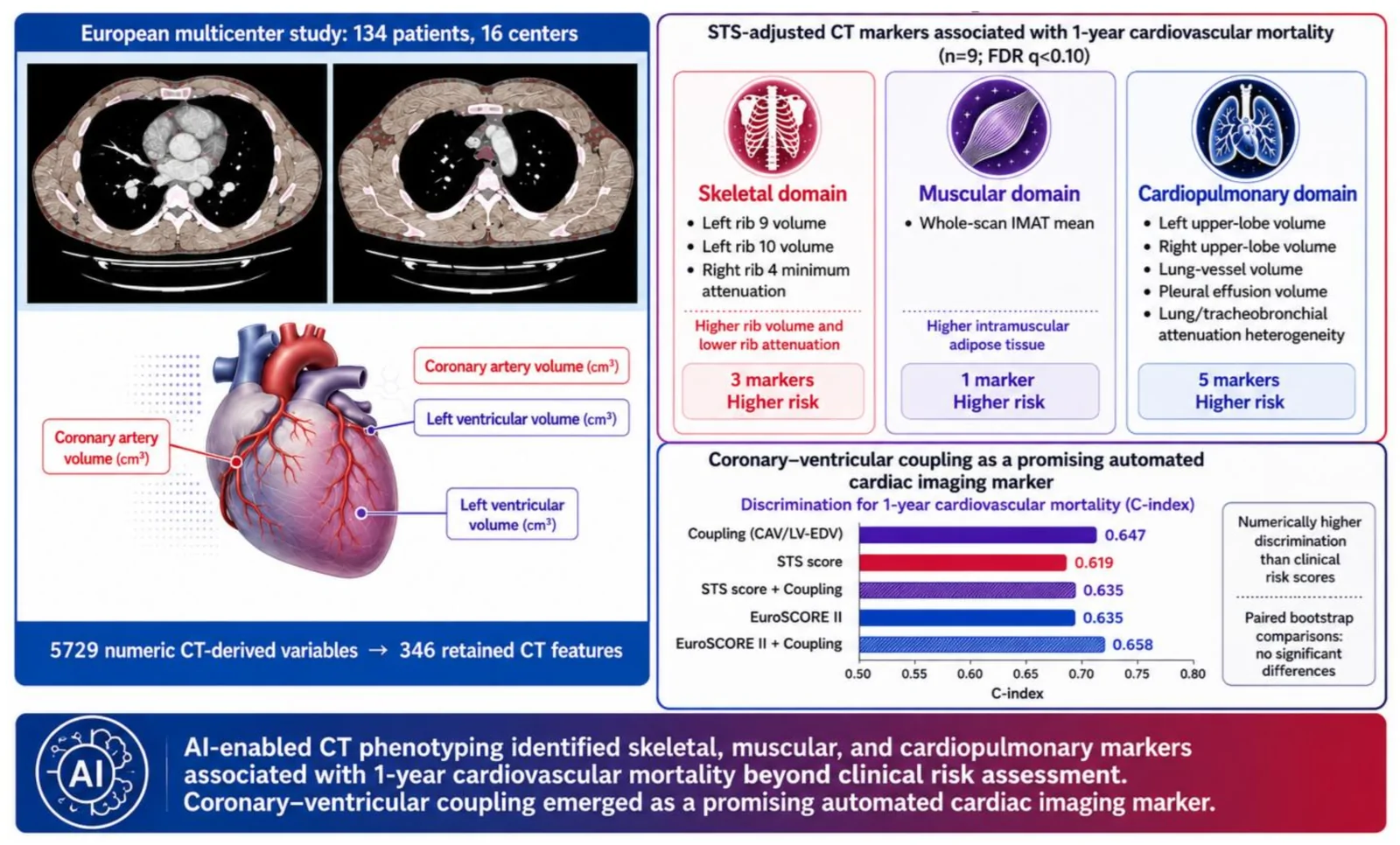
AI-derived CT Phenotyping for Risk Stratification in Transcatheter Mitral Valve Replacement.

**Table 1:** Baseline characteristics of the study population. Continuous variables are reported as median [interquartile range] or mean ± standard deviation. Categorical variables are presented as counts and percentages. STS, Society of Thoracic Surgeons; NT-proBNP, N-terminal pro–B-type natriuretic peptide; TAPSE, tricuspid annular plane systolic excursion; MR, mitral regurgitation; NYHA, New York Heart Association.

Variable	Value	Missing, n
**Continuous variables**		
Age (years)	77.0 [70.0–81.8]	0
Body mass index (kg/m^2^)	25.4 [22.7–28.1]	4
STS score (%)	6.0 [3.5–9.4]	8
EuroSCORE II (%)	6.3 [3.6–10.1]	3
Estimated glomerular filtration rate (ml/min)	42.0 [31.0–57.3]	0
Previous heart failure hospitalizations (n)	1.0 [0.0–1.0]	0
Time from first HF hospitalization to TMVR (months)	3.0 [2.0–5.0]	43
NT-proBNP (pg/mL)	3621.5 [1802.0–7009.2]	23
Left ventricular ejection fraction (%)	48.0 [37.0–56.0]	13
Left ventricular end-diastolic diameter (mm)	54.7 ± 7.8	4
Left ventricular end-systolic diameter (mm)	41.7 ± 9.6	63
Effective regurgitant orifice area (cm^2^)	0.4 [0.3–0.5]	38
Vena contracta (mm)	8.0 [7.0–8.0]	59
Mitral valve gradient (mmHg)	3.0 [2.0–5.0]	29
TAPSE (mm)	17.7 ± 4.2	30
Right ventricle/right atrium gradient (mmHg)	42.9 [32.1–52.0]	44
Central venous pressure (mmHg)	10.0 [5.0–10.0]	47
Systolic pulmonary artery pressure (mmHg)	52.9 ± 13.8	39
**Categorical variables**		
Male sex	76 (56.7%)	0
Diabetes mellitus	34 (25.4%)	0
Hypertension	101 (75.4%)	0
Coronary artery disease	81 (60.4%)	0
Previous myocardial infarction	24 (17.9%)	0
Previous percutaneous coronary intervention	45 (33.6%)	0
Previous Coronary artery bypass grafting	34 (25.4%)	0
Previous stroke	19 (14.2%)	1
Chronic obstructive pulmonary disease	24 (17.9%)	0
Atrial fibrillation/flutter	85 (63.4%)	0
Cardiac implantable electronic device	29 (21.6%)	0
**Previous valve interventions**		
Aortic valve replacement (AVR)	30 (22.4%)	0
Transcatheter aortic valve replacement (TAVR)	14 (10.4%)	0
Both AVR and TAVR	2 (1.5%)	0
Mitral valve replacement	2 (1.5%)	—
Surgical mitral valve repair	2 (1.5%)	—
Mitra clip	9 (6.7%)	—
Annuloplasty	4 (3.0%)	—
**Heart failure status and mitral regurgitation**		
NYHA class I	1 (0.7%)	0
NYHA class II	18 (13.4%)	0
NYHA class III	105 (78.4%)	0
NYHA class IV	10 (7.5%)	0
MR grade ≤ 2	6 (4.4%)	0
MR grade 3	48 (35.8%)	0
MR grade ≥ 4	80 (59.7%)	0
Primary MR	52 (38.8%)	5
Secondary MR	52 (38.8%)	5
Mixed MR	25 (18.7%)	5

MR etiology was missing in 5 patients (3.7%).

**Table 2:** AI-derived CT features meeting the 10% FDR discovery threshold for 1-year cardiovascular mortality. (a). STS-adjusted high-dimensional Cox analysis; (b). CT-only high-dimensional Cox analysis.

Domain	AI-CT Predictor	n	Events	HR/IQR	95% CI	*p*	q
(**a**)							
Skeletal	Total left ninth-rib volume (mL)	83	14	5.56	2.06–14.99	<0.001	0.095
Skeletal	Total left tenth-rib volume (mL)	78	14	3.29	1.52–7.09	0.002	0.095
Skeletal	Right fourth-rib minimum attenuation (HU)	75	13	0.45	0.26–0.75	0.003	0.095
Cardiopulmonary	Lung/tracheobronchial attenuation heterogeneity (SD HU)	94	19	0.42	0.24–0.71	0.001	0.095
Cardiopulmonary	Pleural-effusion volume (mL)	98	19	2.42	1.40–4.18	0.002	0.095
Cardiopulmonary	Left upper-lobe volume (mL)	97	19	2.31	1.36–3.93	0.002	0.095
Cardiopulmonary	Lung-vessel volume (mL)	98	19	2.02	1.29–3.16	0.002	0.095
Cardiopulmonary	Right upper-lobe volume (mL)	92	18	2.75	1.44–5.26	0.002	0.095
Muscular	Whole-scan intramuscular adipose tissue (IMAT) mean	98	19	2.37	1.37–4.10	0.002	0.095
(**b**)							
Cardiopulmonary	Lung/tracheobronchial attenuation heterogeneity (SD HU)	101	21	0.44	0.28–0.70	<0.001	0.087
Cardiopulmonary	Left upper-lobe volume (mL)	103	21	2.38	1.43–3.96	<0.001	0.092
Skeletal	Total left ninth-rib volume (mL)	88	14	5.19	2.05–13.16	<0.001	0.087

Hazard ratios (HRs) are scaled per interquartile-range (IQR) increase. q-values were calculated using the Benjamini–Hochberg false discovery rate procedure separately within each endpoint and model family; q < 0.10 defined the exploratory discovery threshold. No feature met q < 0.10 for 1-year all-cause mortality or in the EuroSCORE II-adjusted high-dimensional analysis. The feature-selection pipeline from 5729 numeric CT-derived variables to 346 retained features is shown in Supplementary Methods S2, Figure S1.

**Table 3:** Coronary–ventricular coupling and 1-year cardiovascular mortality. Primary coupling definition: coronary artery volume (CAV)/left ventricular end-diastolic volume (LV-EDV).

Model	n	Events	HR per IQR		95% CI	*p*	q	C-Index
**A. Association of primary coronary–ventricular coupling with 1-year cardiovascular mortality**								
CT only	86	17	0.47		0.22–1.00	0.049	0.096	0.647
STS-adjusted	79	15	0.49		0.23–1.05	0.067	0.089	0.650
EuroSCORE II-adjusted	83	17	0.47		0.22–0.99	0.046	0.083	0.694
**Model**	**n**	**Events**	**Apparent C-index**		**Optimism-corrected C-index**			**Bootstrap 95% CI**
**B. Bootstrap-based internal validation of discrimination (500 resamples)**								
Coupling	86	17	0.647		0.620			0.391–0.783
STS	79	15	0.619		0.586			0.414–0.749
STS + Coupling	79	15	0.635		0.588			0.446–0.780
EuroSCORE II	83	17	0.635		0.620			0.505–0.758
EuroSCORE II + Coupling	83	17	0.658		0.637			0.550–0.790
**Comparison**			**n**	**Events**	**ΔC**	**95% bootstrap CI**		***p***
**C. Paired bootstrap comparisons of discrimination**								
Coupling vs. STS			79	15	+0.026	−0.189 to 0.238		0.808
STS + Coupling vs. STS			79	15	+0.040	−0.046 to 0.221		0.588
Coupling vs. EuroSCORE II			83	17	+0.017	−0.192 to 0.192		0.836
EuroSCORE II + Coupling vs. EuroSCORE II			83	17	+0.036	−0.016 to 0.180		0.432

Abbreviations: CAV, coronary artery volume; CI, confidence interval; HR, hazard ratio; IQR, interquartile range; LV-EDV, left ventricular end-diastolic volume; STS, Society of Thoracic Surgeons. Nominal *p*-values are reported for the primary CAV/LV-EDV association. q-values reflect Benjamini-Hochberg adjustment across the broader targeted coronary/coupling family and are presented as a supportive multiplicity analysis. Bootstrap confidence intervals and paired ΔC comparisons used 500 resamples. Sample sizes differ according to availability of the coupling measure and the respective clinical risk score. No paired bootstrap comparison was statistically significant.

## Data Availability

Deidentified data may be made available upon reasonable request, subject to approval by the participating institutions.

## Supplementary Materials

The following supporting information can be downloaded at: https://www.mdpi.com/article/10.3390/jcm15197400/s1, Supplementary Results S1—Table S1: AI-derived CT features associated with 1-year cardiovascular mortality; Table S2: STS-adjusted sensitivity analysis of coronary artery volume and cardiovascular mortality; Table S3: Targeted coronary–ventricular coupling analysis; Table S4: Alternative CAV normalization strategies and 1-year cardiovascular mortality; Table S5: Sensitivity analysis of identified CT phenotypes in relation to baseline MR severity; Table S6: Outcome-independent domain PCA and penalized multidomain modelling. Supplementary Methods S2: Extended Imaging Harmonization and Statistical Analysis—Figure S1 Patient and imaging flow (A) and High-dimensional CT feature-selection pipeline (B).

## Abbreviations

The following abbreviations are used in this manuscript:

Abbreviation	Definition
AI	Artificial intelligence
AVR	Aortic valve replacement
BH	Benjamini–Hochberg
BOA	Body and organ analysis
CAV	Coronary artery volume
CCTA	Coronary computed tomography angiography
CI	Confidence interval
CT	Computed tomography
ECG	Electrocardiogram/electrocardiographic
EuroSCORE II	European System for Cardiac Operative Risk Evaluation II
FDR	False discovery rate
HF	Heart failure
HR	Hazard ratio
HU	Hounsfield units
IMAT	Intramuscular adipose tissue
IQR	Interquartile range
LV	Left ventricle/left ventricular
LV-EDV	Left ventricular end-diastolic volume
LVEDDi	Left ventricular end-diastolic diameter index
LVOT	Left ventricular outflow tract
MACE	Major adverse cardiovascular events
MR	Mitral regurgitation
NT-proBNP	N-terminal pro-B-type natriuretic peptide
NYHA	New York Heart Association
PCA	Principal component analysis
SD	Standard deviation
STS	Society of Thoracic Surgeons
STS-PROM	Society of Thoracic Surgeons Predicted Risk of Mortality
TAPSE	Tricuspid annular plane systolic excursion
TAVR	Transcatheter aortic valve replacement
TMVR	Transcatheter mitral valve replacement
