# Supplementary material for: Artificial Intelligence-Derived Computed Tomography Phenotyping for Cardiovascular Risk Stratification in Transcatheter Mitral Valve Replacement
Source: J Clin Med. 2026 Sep 23;15(19):7400. doi: 10.3390/jcm15197400 (PMC13665488; doi:10.3390/jcm15197400)
Supplement: Supplementary file 1 [file jcm-15-07400-s001.zip › new-Supplementary_Methods_S2_TendyneAI_210926-0044.pdf]

Supplementary Methods S2
Extended Imaging Harmonization and Statistical Analysis

Artificial Intelligence–Derived Computed Tomography Phenotyping for Risk Stratification in Transcatheter Mitral Valve Replacement

S2.1 Study cohort and imaging availability

The final clinical analysis cohort comprised 134 TENDER registry patients. Imaging files were available on disk for 139 patients across 16 European centers; 138/139 had a selected scan suitable for Body and Organ Analysis (BOA), and 128/139 had a usable multiphase cardiac acquisition. After clinical–imaging matching, 133/134 clinical patients had usable imaging information. One patient had clinical data but no usable imaging. Feature-level sample size therefore varied with field of view, structure coverage, endpoint availability, and clinical-score completeness.

Item	Final count / definition
Clinical analysis cohort	134
Centres contributing imaging	16
Patients on imaging archive	139
BOA scan selected	138/139
Usable cardiac acquisition	128/139
Clinical rows with imaging	133/134
Cardiac phase position genuinely labelled in R-R interval	84/133 in final harmonized dataset; otherwise phase percentage was ordinal only

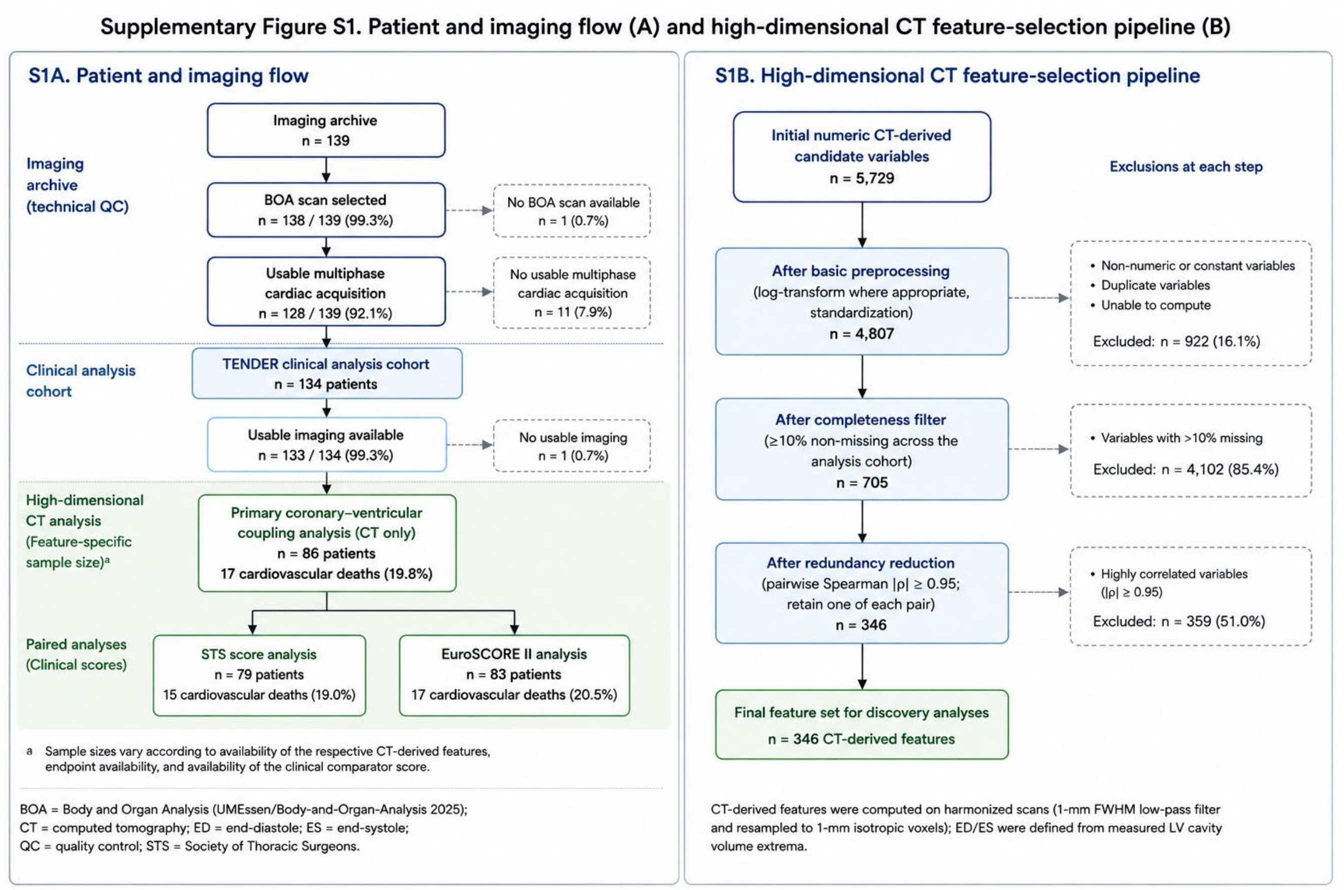

Figure S1A/B Patient and imaging flow / High-dimensional CT feature-selection pipeline

S2.2 Scan selection and harmonization

A geometry-based scan-selection procedure was used to avoid treating a packed multiphase cardiac acquisition as a large anatomical scan. Z-axis anatomical coverage was measured directly from DICOM ImagePositionPatient information rather than calculated as slice count multiplied by slice thickness. Series were screened for readability, three-dimensional geometry, image-plane size, sufficient slice number and anatomical coverage. For BOA, the largest suitable three-dimensional volume for each patient was selected, with preference for a soft reconstruction kernel when otherwise comparable. Cardiac analysis used all separable phases from a single selected acquisition so that end-diastolic and end-systolic measurements were derived within the same field of view.

Every selected BOA scan and cardiac phase volume was harmonized to the same target used in the related surgical and TAVI imaging studies: Gaussian low-pass filtering to 1 mm full width at half maximum (FWHM), resampling to a 1 mm isotropic grid, and storage as int16 with Hounsfield-unit clamping.

S2.3 Cardiac phase definition

No scanner-provided phase label was used to define end-diastole (ED) or end-systole (ES). Instead, all available phases of the selected cardiac acquisition were segmented, and ED/ES were defined as the measured maximum/minimum of the left-ventricular cavity. The same two phases were then used for the other cardiac structures. This was chosen because scanner phase labels identified the exact measured LV extreme in only approximately half of checkable acquisitions and many centres provided no interpretable R-R position. The phase-volume measurements remained usable even when the phase percentage was ordinal rather than physiological.

S2.4 Automated CT feature generation

Automated phenotyping used BOA and Comp2Comp outputs together with harmonized phase-resolved cardiac segmentations. Candidate measurements included organ and tissue volumes, attenuation summaries, body-composition measurements, skeletal features, vascular measurements, pulmonary/airway measurements, effusion measures, and cardiac chamber/myocardial volumes. Agatston scores were excluded because the cohort consisted of contrast-enhanced planning CT angiography, in which standard fixed-threshold Agatston scoring is not valid; volume-based calcium outputs remained eligible where available.

S2.5 Endpoint definition and quality control

Patient-level endpoint quality control verified event classification within the prespecified time windows. The final endpoint totals were 34 all-cause and 21 cardiovascular deaths within one year, and 9 all-cause and 7 cardiovascular deaths within 30 days. Cardiovascular mortality was classified according to Mitral Valve Academic Research Consortium (MVARC) endpoint definitions[1]. Validated Kaplan–Meier analysis time was available for all 134 patients; median observed analysis time was 365 days (IQR 258.5–365 days), with administrative censoring at 365 days.

Endpoint	Usable n	Events
1-year all-cause mortality	114	34
1-year cardiovascular mortality	105	21
30-day all-cause mortality	125	9
30-day cardiovascular mortality	114	7

Thirty-day cardiovascular and all-cause mortality were explored descriptively; with only 7 and 9 events, respectively, event counts were considered insufficient for meaningful high-dimensional inference.

S2.6 High-dimensional CT discovery analysis

The discovery universe was restricted to numeric automated CT measurements. Clinical registry variables were not eligible as imaging predictors and were used only for endpoint definition, clinical comparators (STS and EuroSCORE II), and dedicated body-size or sensitivity analyses. Before any outcome modelling, presence/segmentation flags, technical acquisition and bookkeeping variables, invalid Agatston variables, and all known outcome/follow-up leakage variables were removed.

For the remaining variables, feature availability, number of unique observed values, range and skewness were assessed. Features required at least three unique observed values and at least 75% completeness. Pronounced right-skewness (skewness >1) was handled by log transformation for strictly positive variables or log1p transformation for non-negative variables

containing zero. Redundancy was then reduced using pairwise Spearman correlation on the transformed analysis scale. At |ρ|≥0.95, a deterministic outcome-blind rule retained the variable with higher completeness, then more unique values, then alphabetically first in the event of a remaining tie.

Feature-selection step	Number of variables
Numeric automated CT candidates after metadata/clinical exclusions	5729
≥3 unique observed values	4807
≥75% completeness	705
Removed by |Spearman ρ|≥0.95	359
Final discovery-screen variables	346

Each of the 346 features was tested separately for 1-year cardiovascular and all-cause mortality using Cox proportional-hazards models in three families: CT-only, CT+STS, and CT+EuroSCORE II. Hazard ratios were reported per interquartile-range (IQR) increase. Benjamini–Hochberg FDR correction was applied separately within each endpoint-by-adjustment family. Given the exploratory high-dimensional purpose of the screen, BH-FDR q<0.10 was used as the discovery threshold. This threshold describes the final analysis interpretation and is not labelled as prespecified.

S2.7 Discovery results used in the manuscript

For 1-year cardiovascular mortality, three CT-only features and nine STS-adjusted features met q<0.10. No high-dimensional feature met q<0.10 after EuroSCORE II adjustment. No high-dimensional feature met q<0.10 for 1-year all-cause mortality in any adjustment family.

Model	Feature	n	Events	HR/IQR	95% CI	q
CT only	Lung/tracheobronchial attenuation heterogeneity	101	21	0.44	0.28–0.70	0.087
CT only	Total left rib 9 volume	88	14	5.19	2.05–13.16	0.087
CT only	Left upper-lobe volume	103	21	2.38	1.43–3.96	0.092
CT + STS	Total left rib 9 volume	83	14	5.56	2.06–14.99	0.095
CT + STS	Lung/tracheobronchial attenuation heterogeneity	94	19	0.42	0.24–0.71	0.095
CT + STS	Pleural effusion volume	98	19	2.42	1.40–4.18	0.095
CT + STS	Whole-scan intramuscular adipose tissue (IMAT) mean	98	19	2.37	1.37–4.10	0.095
CT + STS	Left upper-lobe volume	97	19	2.31	1.36–3.93	0.095
CT + STS	Lung-vessel volume	98	19	2.02	1.29–3.16	0.095
CT + STS	Right upper-lobe volume	92	18	2.75	1.44–5.26	0.095
CT + STS	Total left rib 10 volume	78	14	3.29	1.52–7.09	0.095
CT + STS	Right rib 4 minimum attenuation	75	13	0.45	0.26–0.75	0.095

S2.8 Targeted coronary and coronary–ventricular coupling analysis

A separate targeted mechanistic family was analysed independently of the 346-feature discovery screen. The source variables were raw CAV, LV-ED volume, LV-ES volume, myocardial-ED volume, and myocardial-ES volume. Four raw ratios were calculated directly from these biological volumes: CAV/LV-ED, CAV/LV-ES, CAV/myocardium-ED, and CAV/myocardium-ES. These targeted variables were not subjected to the high-dimensional skewness transformation; HRs

therefore refer to an IQR increase on the raw physical ratio scale. This distinction is important when comparing effect estimates with transformed variables from the discovery pipeline.

Cox models were fitted for 1-year cardiovascular and all-cause mortality as CT-only, +STS and +EuroSCORE II analyses. **CAV/LV-EDV was defined as the primary anatomically motivated coupling measure. Alternative normalization using LV-ES volume and myocardial ED/ES volumes, as well as CAV alone, was evaluated as supportive sensitivity analysis. Nominal p-values are reported for the primary CAV/LV-EDV association. In addition, BH-FDR-adjusted q-values were calculated within each targeted endpoint-by-adjustment family as a supportive multiplicity analysis.** Natural splines with 3 degrees of freedom were compared with linear models using likelihood-ratio tests, with separate BH correction for spline overall association and nonlinearity.

Adjustment	Targeted feature	n	Events	HR/IQR	95% CI	p	q
CT only	CAV / Myocardium-ES	85	17	0.42	0.18–0.96	0.040	0.096
CT only	CAV / LV-ES	86	17	0.40	0.17–0.97	0.043	0.096
CT only	CAV / Myocardium-ED	86	17	0.45	0.21–0.98	0.044	0.096
CT only	CAV / LV-ED	86	17	0.47	0.22–1.00	0.049	0.096
CT only	CAV	97	20	0.51	0.26–1.01	0.053	0.096
+STS	Myocardial ED volume	87	16	1.99	1.02–3.90	0.044	0.089
+STS	Myocardial ES volume	86	16	1.88	1.01–3.50	0.047	0.089
+STS	CAV / Myocardium-ES	78	15	0.44	0.19–1.02	0.056	0.089
+STS	CAV / LV-ES	79	15	0.42	0.17–1.04	0.060	0.089
+STS	CAV / Myocardium-ED	79	15	0.48	0.22–1.04	0.062	0.089
+STS	CAV / LV-ED	79	15	0.49	0.23–1.05	0.067	0.089
+STS	CAV	90	18	0.53	0.26–1.05	0.069	0.089
+EuroSCORE II	CAV	94	20	0.49	0.25–0.95	0.035	0.083
+EuroSCORE II	CAV / Myocardium-ES	82	17	0.42	0.19–0.96	0.039	0.083
+EuroSCORE II	CAV / Myocardium-ED	83	17	0.45	0.21–0.97	0.041	0.083
+EuroSCORE II	CAV / LV-ES	83	17	0.41	0.17–0.98	0.045	0.083
+EuroSCORE II	CAV / LV-ED	83	17	0.47	0.22–0.99	0.046	0.083

In the final targeted spline analysis, no cardiovascular nonlinearity test met q<0.10. No targeted coronary/coupling variable met q<0.10 for 1-year all-cause mortality. Accordingly, the manuscript emphasizes the targeted linear raw-ratio findings and treats them as hypothesis-generating rather than as a validated replacement for a clinical risk score.

S2.9 Outcome-independent domain PCA

The 346-feature set retained after preprocessing was reused without additional feature filtering for PCA. A complete baseline-variable blacklist was checked again. Features were assigned to Coronary, Cardiac, Vascular, Lung/Airway, Effusions, Skeletal, Body Composition and Other Organs domains; two transformed coupling variables were added as a separate Coupling domain. Missing values were median-imputed within variable for PCA only. PCA was performed after centring and scaling. The minimum number of PCs required to reach 70% cumulative variance was retained, with a maximum of three PCs per domain; if 70% was not reached by three PCs, three were retained. PCA outcome results are reported in Supplementary Results S1, Table S6.

S2.10 Penalized multidomain score and internal validation

Elastic-net Cox regression used $\alpha=0.5$ and the cross-validated λ1se penalty. Domain PCs were penalized; STS or EuroSCORE II, when included, were unpenalized. Models were run with and without the Coupling domain. Two patients had day-0 event times; these times were changed to 0.5 days only for glmnet, which requires strictly positive survival time, while all standard Cox analyses retained the original times. Bootstrap optimism correction used 200 repetitions and repeated penalized tuning in each bootstrap sample. Penalized-model results are reported in Supplementary Results S1, Table S6.

S2.11 Additional sensitivity analyses

The final analysis package retained proportional-hazards diagnostics, calibration summaries, C-index estimates, paired bootstrap C-index comparisons for the transformed ED coupling definitions, competing-risk Fine–Gray analyses for cardiovascular death, Kaplan–Meier visualizations, and a CABG sensitivity analysis for CAV. These analyses were considered supportive rather than primary. In the competing-risk models, transformed CAV/LV-ED and CAV/myocardium-ED ratios were not significantly associated with cardiovascular death. Excluding patients with prior CABG did not materially change the direction of the CAV association, and there was no evidence of a CAV-by-CABG interaction. These sensitivity findings reinforce the exploratory nature of the targeted coronary signal.

A post hoc MR-severity sensitivity analysis was additionally performed. Baseline MR grade (1-4) was treated as an ordinal variable and was first evaluated in Cox proportional-hazards models for 1-year all-cause and cardiovascular mortality, both unadjusted and after adjustment for STS-PROM. For the nine CT features that met $q<0.10$ in the STS-adjusted 1-year cardiovascular-mortality discovery analysis, Spearman rank correlations were then calculated between MR grade and each selected CT feature. Each feature was subsequently re-fitted in a Cox proportional-hazards model including STS-PROM and baseline MR grade, using the same 1-year cardiovascular endpoint and IQR scaling as the primary analysis. Benjamini-Hochberg correction was applied across the nine selected features for the correlation tests and separately across the nine MR-adjusted Cox models. Results are reported in Supplementary Results S1, Table 5.

S2.12 Reproducibility of the analysis workflow

The analysis workflow comprised three linked components: (1) high-dimensional FDR discovery, which generated the endpoint-resolved analysis dataset and post-correlation feature set; (2) outcome-independent domain PCA and penalized multidomain modelling, which used the same retained feature set; and (3) the targeted coronary/coupling component analysis, which used the same analysis dataset. No outcome-dependent feature selection was performed before the discovery Cox screen or PCA.

S2.13 Procedural characteristics - Supplementary Table

Variable	Value	Available n	Missing n
**Tendyne device size**		**99**	**35**

24 mm	1 (1.0%)		
27 mm	5 (5.1%)		
29 mm	32 (32.3%)		
33 mm	28 (28.3%)		
35 mm	22 (22.2%)		
37 mm	9 (9.1%)		
39 mm	1 (1.0%)		
41 mm	1 (1.0%)		
**Successful implantation**	**131 (98.5%)**	**133**	**1**
**Residual mitral regurgitation after TMVR**		**133**	**1**
None (grade 0)	128 (96.2%)		
Mild (grade 1/1+)	3 (2.3%)		
Grade 2	1 (0.8%)		
Grade 3	1 (0.8%)		
Grade 4	0 (0.0%)		
**LVOT obstruction**	**4 (3.0%)**	**134**	**0**
**Postprocedural bleeding, BARC 2/3/5**	**19 (14.2%)**	**134**	**0**
**In-hospital length of stay, days**	**12.0 [9.0-18.8]**	**130**	**4**

Data are presented as n (%) or median [interquartile range]. Percentages are calculated using the number of patients with available data for the respective variable. Device-size entries with differing registry notation were harmonized according to the nominal device diameter. BARC, Bleeding Academic Research Consortium; LVOT, left ventricular outflow tract; TMVR, transcatheter mitral valve replacement.

REFERENCES:

1) Stone, G, Adams, D, Abraham, W. et al. Clinical Trial Design Principles and Endpoint Definitions for Transcatheter Mitral Valve Repair and Replacement: Part 2: Endpoint Definitions: A Consensus Document From the Mitral Valve Academic Research Consortium. JACC. 2015 Jul, 66 (3) 308–321. https://doi.org/10.1016/j.jacc.2015.05.049
