# Supplementary material for: Artificial Intelligence-Derived Computed Tomography Phenotyping for Cardiovascular Risk Stratification in Transcatheter Mitral Valve Replacement
Source: J Clin Med. 2026 Sep 23;15(19):7400. doi: 10.3390/jcm15197400 (PMC13665488; doi:10.3390/jcm15197400)
Supplement: Supplementary file 1 [file jcm-15-07400-s001.zip › Supplementary_Results_S1_TendyneAI_n.pdf]

SUPPLEMENTARY RESULTS S1

Table S1. AI-derived CT features associated with 1-year cardiovascular mortality

Table S1 A. STS-adjusted high-dimensional Cox analysis

Domain	AI-CT predictor	n	Events	HR/IQR	95% CI	p	q	C-index
Skeletal	Total rib volume, left rib 9 (mL)	83	14	5.56	2.06–14.99	<0.001	0.095	0.778
Cardiopulmonary	Lung/tracheobronchial attenuation heterogeneity (SD HU)	94	19	0.42	0.24–0.71	0.001	0.095	0.672
Cardiopulmonary	Pleural effusion volume (mL)	98	19	2.42	1.40–4.18	0.002	0.095	0.670
Muscular	Whole-scan intramuscular adipose tissue (IMAT) mean	98	19	2.37	1.37–4.10	0.002	0.095	0.660
Cardiopulmonary	Left upper-lobe volume (mL)	97	19	2.31	1.36–3.93	0.002	0.095	0.667
Cardiopulmonary	Lung-vessel volume (mL)	98	19	2.02	1.29–3.16	0.002	0.095	0.687
Cardiopulmonary	Right upper-lobe volume (mL)	92	18	2.75	1.44–5.26	0.002	0.095	0.678
Skeletal	Total rib volume, left rib 10 (mL)	78	14	3.29	1.52–7.09	0.002	0.095	0.753
Skeletal	Right rib 4 minimum attenuation (HU)	75	13	0.45	0.26–0.75	0.003	0.095	0.710

Table S1 B. CT-only high-dimensional Cox analysis

Domain	AI-CT predictor	n	Events	HR/IQR	95% CI	p	q	C-index
Cardiopulmonary	Lung/tracheobronchial attenuation heterogeneity (SD HU)	101	21	0.44	0.28–0.70	<0.001	0.087	0.697
Skeletal	Total rib volume, left rib 9 (mL)	88	14	5.19	2.05–13.16	<0.001	0.087	0.790
Cardiopulmonary	Left upper-lobe volume (mL)	103	21	2.38	1.43–3.96	<0.001	0.092	0.685

Hazard ratios are scaled per interquartile-range increase. q-values are Benjamini–Hochberg FDR-adjusted within each endpoint and model family; q<0.10 was the exploratory discovery threshold. No feature met q<0.10 for 1-year all-cause mortality or in the EuroSCORE II-adjusted high-dimensional analysis. The feature-selection pipeline (5,729 numeric CT-derived variables to 346 retained features) is detailed in Supplementary Methods S2.

Table S2. STS-adjusted sensitivity analysis of coronary artery volume and cardiovascular mortality

Cohort / term	n	Events	HR/IQR	95% CI	p-value
All patients	90	18	0.54	0.28–1.05	0.069
CABG excluded	67	15	0.51	0.24–1.11	0.089
CAV × CABG interaction	90	18	1.68	0.30–9.33	0.552

Models were adjusted for STS score. The CABG-excluded analysis assessed robustness of the CAV association to prior coronary bypass surgery; the interaction term tested effect modification by CABG status.

Table S3. Targeted coronary–ventricular coupling analysis

Table S3 A. Primary CAV/LV-EDV coupling measure for 1-year cardiovascular mortality

Model	n	Events	HR/IQR	95% CI	p	q	C-index
CT only	86	17	0.47	0.22–1.00	0.049	0.096	0.647
+STS	79	15	0.49	0.23–1.05	0.067	0.089	0.650
+EuroSCORE II	83	17	0.47	0.22–0.99	0.046	0.083	0.694

Coronary–ventricular coupling was defined as coronary artery volume (CAV) divided by left ventricular end-diastolic volume (LV-EDV). LV-EDV corresponded to the maximal segmented LV cavity volume. q-values reflect BH-FDR correction within the targeted coronary/coupling analysis family.

Table S3 B. Bootstrap comparisons of discrimination for the primary coupling measure

Comparison	n	Events	Δ C-index	95% bootstrap CI	p-bootstrap
CAV/LV-EDV vs STS	79	15	+0.026	-0.189 to +0.238	0.808
CAV/LV-EDV vs EuroSCORE II	83	17	+0.017	-0.192 to +0.192	0.836
STS+CAV/LV-EDV vs STS	79	15	+0.040	-0.046 to +0.221	0.588
EuroSCORE II+CAV/LV-EDV vs EuroSCORE II	83	17	+0.036	-0.016 to +0.180	0.432

Paired bootstrap comparisons used the same complete-case subset for each model comparison (500 resamples). Positive Δ C-index indicates numerically higher discrimination for the model containing or represented by CAV/LV-EDV. None of the formal bootstrap comparisons was statistically significant.

Table S3 C. Alternative normalization and component analyses (CT-only)

Feature	n	Events	HR/IQR	95% CI	p	q	C-index
CAV / LV-ES	86	17	0.40	0.17–0.97	0.043	0.096	0.662
CAV / Myocardium-ED	86	17	0.45	0.21–0.98	0.044	0.096	0.649
CAV / Myocardium-ES	85	17	0.42	0.18–0.96	0.040	0.096	0.657
Coronary artery volume	97	20	0.51	0.26–1.01	0.053	0.096	0.636
LV end-diastolic volume	94	18	1.30	0.78–2.19	0.313	0.313	0.562

Alternative coupling ratios showed directionally consistent inverse associations. The targeted analysis also examined CAV and the individual cardiac-volume components separately. Full STS- and EuroSCORE II-adjusted component results, spline analyses, calibration, competing-risk analyses, and additional sensitivity analyses are reported in Supplementary Methods S2.

Abbreviations: BH-FDR, Benjamini–Hochberg false discovery rate; CAV, coronary artery volume; CABG, coronary artery bypass grafting; CT, computed tomography; HR, hazard ratio; IQR, interquartile range; LV-EDV, left ventricular end-diastolic volume; LV-ESV, left ventricular end-systolic volume; STS, Society of Thoracic Surgeons.

Supplementary Table S4. Alternative CAV normalization strategies and 1-year cardiovascular mortality

Primary CAV/LV-EDV coupling is shown with ventricular and myocardial sensitivity definitions and CAV alone.

Coupling / component	n	Events	HR per IQR	95% CI	p	q	C-index
**A. CT-only models**							
CAV / LV-EDV (primary)	86	17	0.47	0.22-1.00	0.049	0.096	0.647
CAV / LV-ESV	86	17	0.40	0.17-0.97	0.043	0.096	0.662
CAV / myocardial ED volume	86	17	0.45	0.21-0.98	0.044	0.096	0.649
CAV / myocardial ES volume	85	17	0.42	0.18-0.96	0.040	0.096	0.657
CAV alone	97	20	0.51	0.26-1.01	0.053	0.096	0.636
**B. STS-adjusted models**							
CAV / LV-EDV (primary)	79	15	0.49	0.23-1.05	0.067	0.089	0.650
CAV / LV-ESV	79	15	0.42	0.17-1.04	0.060	0.089	0.668
CAV / myocardial ED volume	79	15	0.48	0.22-1.04	0.062	0.089	0.659
CAV / myocardial ES volume	78	15	0.44	0.19-1.02	0.056	0.089	0.669
CAV alone	90	18	0.53	0.26-1.05	0.069	0.089	0.632
**C. EuroSCORE II-adjusted models**							
CAV / LV-EDV (primary)	83	17	0.47	0.22-0.99	0.046	0.083	0.694
CAV / LV-ESV	83	17	0.41	0.17-0.98	0.045	0.083	0.704
CAV / myocardial ED volume	83	17	0.45	0.21-0.97	0.041	0.083	0.692
CAV / myocardial ES volume	82	17	0.42	0.19-0.96	0.039	0.083	0.704
CAV alone	94	20	0.49	0.25-0.95	0.035	0.083	0.675

Abbreviations: CAV, coronary artery volume; ED, end-diastolic; ES, end-systolic; ESV, end-systolic volume; HR, hazard ratio; IQR, interquartile range; LV, left ventricular; STS, Society of Thoracic Surgeons. Ratios used raw CT-derived volumes. q-values were BH-adjusted within each targeted model family. CAV/LV-EDV was primary; other denominators were sensitivity analyses.

Supplementary Table S5. Sensitivity analysis of identified CT phenotypes in relation to baseline MR severity

Table S5 A. Spearman correlations between baseline MR grade and the nine STS-adjusted cardiovascular-mortality discovery features

AI-CT predictor	n	Spearman rho	p	q
Total left ninth-rib volume (mL)	93	0.225	0.0301	0.0338
Lung/tracheobronchial attenuation heterogeneity (SD HU)	109	-0.269	0.00464	0.00596
Pleural-effusion volume (mL)	113	0.267	0.0043	0.00596
Whole-scan intramuscular adipose tissue (IMAT) mean	114	0.171	0.0682	0.0682
Left upper-lobe volume (mL)	111	0.288	0.00215	0.00596
Lung-vessel volume (mL)	113	0.268	0.00414	0.00596
Right upper-lobe volume (mL)	107	0.317	0.000868	0.00524
Total left tenth-rib volume (mL)	85	0.318	0.00306	0.00596
Right fourth-rib minimum attenuation (HU)	85	-0.346	0.00116	0.00524

Table S5 B. One-year cardiovascular mortality after additional adjustment for STS-PROM and baseline MR grade

AI-CT predictor	n	Events	HR/IQR	95% CI	p	q
Total left ninth-rib volume (mL)	71	12	6.07	1.80-20.43	0.00359	0.0322
Total left tenth-rib volume (mL)	66	12	3.76	1.43-9.85	0.00715	0.0322
Whole-scan intramuscular adipose tissue (IMAT) mean	84	17	1.93	1.07-3.50	0.0295	0.0683
Lung-vessel volume (mL)	84	17	1.88	1.05-3.36	0.0326	0.0683
Right fourth-rib minimum attenuation (HU)	64	12	0.52	0.28-0.98	0.0417	0.0683
Right upper-lobe volume (mL)	79	16	2.19	1.02-4.74	0.0455	0.0683
Left upper-lobe volume (mL)	83	17	1.92	0.99-3.71	0.0538	0.0692
Pleural-effusion volume (mL)	84	17	1.81	0.95-3.46	0.0712	0.0801
Lung/tracheobronchial attenuation heterogeneity (SD HU)	81	17	0.49	0.21-1.14	0.0974	0.0974

Table S5 C. Association of baseline MR grade with 1-year mortality

**Endpoint**	**Model**	**n**	**Events**	**HR per one-grade increase**	**95% CI**	**p**
1-year all-cause mortality	Unadjusted	95	26	5.56	1.71-18.07	0.004
1-year all-cause mortality	STS-PROM adjusted	92	25	5.15	1.58-16.81	0.007
1-year cardiovascular mortality	Unadjusted	87	17	9.64	1.31-70.71	0.026
1-year cardiovascular mortality	STS-PROM adjusted	84	17	10.12	1.36-75.13	0.024

Baseline MR grade was treated as an ordinal variable (grades 1-4). Table 7A reports Spearman correlations; q-values are Benjamini-Hochberg adjusted across the nine selected CT features. Table 7B reports Cox models for 1-year cardiovascular mortality adjusted for STS-PROM and baseline MR grade, with HRs scaled per IQR increase in each CT feature and BH-FDR correction across this targeted nine-feature sensitivity set. Table 7C reports the association of baseline MR grade itself with 1-year all-cause and cardiovascular mortality, both unadjusted and after STS-PROM adjustment. Because the nine features were selected from the preceding discovery analysis, the post hoc q-values in Tables 7A-B are descriptive sensitivity-analysis measures and should not be interpreted as an independent discovery procedure. MR, mitral regurgitation; STS-PROM, Society of Thoracic Surgeons Predicted Risk of Mortality.

Supplementary Table S6. Outcome-independent domain PCA and penalized multidomain modelling

PCA domain structure, cardiovascular-mortality associations, and bootstrap-validated penalized multidomain models.

Table S6 A. PCA domain structure

**Domain**	**n variables**	**PCs retained**	**Variance explained by retained PCs**
Coupling	2	1	99.9%
Coronary	7	2	82.5%
Body Composition	46	3	66.8%
Effusions	12	3	61.3%
Skeletal	89	3	55.7%
Vascular	16	3	52.8%
Lung/Airway	83	3	48.0%
Cardiac	42	3	48.0%
Other Organs	51	3	38.1%

The minimum number of principal components (PCs) required to reach 70% cumulative variance was retained, with a maximum of three PCs per domain; if 70% was not reached by three PCs, three were retained.

Table S6 B. CT-only PCA components meeting the 10% FDR threshold for 1-year cardiovascular mortality

**Domain / component**	**HR per SD**	**95% CI**	**p**	**q**
Vascular PC3	1.77	1.18–2.66	0.0059	0.089
Other-Organs PC2	0.70	0.54–0.91	0.0074	0.089

Neither component remained below q<0.10 after adjustment for STS or EuroSCORE II. The Coupling-domain PC did not show a significant linear association with outcome on the transformed PCA scale.

Table S6 C. Penalized multidomain score and bootstrap internal validation

**Endpoint / model**	**Apparent C**	**Bootstrap-corrected C**	**Selected predictors**
1-year cardiovascular mortality, CT PCs only	0.500	0.459	None

Endpoint / model	Apparent C	Bootstrap-corrected C	Selected predictors
1-year cardiovascular mortality, STS	0.567	0.555	STS only
1-year cardiovascular mortality, EuroSCORE II	0.594	0.585	EuroSCORE II only
1-year all-cause mortality, CT PCs only	0.500	0.462	None
1-year all-cause mortality, STS	0.567	0.565	STS only
1-year all-cause mortality, EuroSCORE II	0.566	0.560	EuroSCORE II only

At the cross-validated λ1se penalty, the CT-only model selected no CT principal components for either endpoint. In models containing a clinical score, only the unpenalized clinical score was retained. Bootstrap optimism correction used 200 repetitions with repeated penalized tuning in each bootstrap sample.

Abbreviations: CI, confidence interval; FDR, false discovery rate; HR, hazard ratio; PC, principal component; PCA, principal component analysis; SD, standard deviation; STS, Society of Thoracic Surgeons.
